# Graduates of the Veterinary Department, University of Pennsylvania

**Published:** 1887-07

**Authors:** 


### GRADUATES OF THE VETERINARY DEPARTMENT, UNIVERSITY OF PENNSYLVANIA.

At the 131st annual commencement of the University of Pennsylvania, and the first of the Veterinary Department, held in the Academy of Music, Philadelphia, June 8th, 1887, the degree of *Veterinariæ Medicinæ Doctor* was conferred on the following gentlemen, in order of merit as named :

Richard W. Hickman, Bustleton, Pennsylvania,
Simon Harger, Hecktown, Penn.,
Hiram P. Eves, Lima, Penn.,
Charles Williams, Fellowship, New Jersey,
Charles Lintz, Holmesburg, Penn.,
Edgar Marlin, Philadelphia, Pennsylvania,
John F. Vandegrift, Langhorne, Pennsylvania,
William B. Montgomery, Chestnut Hill, Penn.,
Richard G. Webster, Media, Penn.,
Charles Cullen, Philadelphia, Pennsylvania.

---

